# Risk factors for ocular biological parameters in Chinese preschool children: a cohort study from the Beijing whole childhood eye study

**DOI:** 10.3389/fmed.2025.1510124

**Published:** 2025-02-07

**Authors:** Xi Qin, Yunyun Sun, Shana Wang, Xiaolan Xie, Lei Gao, Huijian Li, Ruihua Wei, Jing Fu, Bidan Zhu

**Affiliations:** ^1^Tongzhou Maternal and Child Health Hospital of Beijing, Beijing, China; ^2^Beijing Tongren Eye Center, Beijing Tongren Hospital, Beijing Ophthalmology & Visual Sciences Key Laboratory, Capital Medical University, Beijing, China; ^3^Tianjin Key Laboratory of Retinal Functions and Diseases, Tianjin Branch of National Clinical Research Center for Ocular Disease, Eye Institute and School of Optometry, Tianjin Medical University Eye Hospital, Tianjin, China

**Keywords:** ocular biological parameters, preschool children, parental myopia, eye habits, growth and development

## Abstract

**Background:**

The high myopia prevalence in young East Asian children necessitates early detection and prevention strategies. Axial length (AL), corneal radius of curvature (CR), and the AL to CR ratio (AL/CR) are potential myopia biomarkers. However, the influence of genetic, growth and development, and environmental factors on these metrics in Chinese preschool children remains unclear. Therefore, this study aimed to investigate the effects of these factors on the AL, CR, and AL/CR ratio in children aged 3–6 years in Beijing.

**Methods:**

In this 3-month study, initiated in November 2021, children aged 3–6 years from nine kindergartens in Beijing were randomly selected for ocular biological parameter measurements. The height and weight of each child were measured, and their parents completed a questionnaire on parental myopia and environmental influences. The AL/CR ratio were calculated. One-way analysis of variance, univariate analysis, and multiple linear regression models (with age, sex, height, weight, parental myopia, continuous near-work time, electronic products use, and outdoor activity time as variables) were used to compare the effects of different variables on the AL, CR, and AL/CR ratio.

**Results:**

Overall, 1,353 participants (708 boys; mean age, 4.37 ± 0.82 years) were included in this study. The multiple linear regression analysis revealed that parental myopia significantly increased the AL and AL/CR ratio (*p* = 0.002, *p* < 0.001). Male participants had a longer AL, larger CR, and greater AL/CR ratio than female participants (all *p* < 0.001). A longer AL and larger CR were associated with greater height (both *p* < 0.001). The AL/CR ratio increased with age (*p* < 0.001). The CR was positively associated with the amount of time spent outdoors (*p* = 0.004).

**Conclusion:**

Ocular biological parameters are influenced by genetic, growth and development, and environmental factors. Among children aged 3–6 years in Beijing, monitoring growth and development, investigating parental myopia, and evaluating eye habits have certain guiding significance for delaying increases in the AL and AL/CR ratio. This study may provide some suggestions for the development of healthy eye habits.

## 1 Background

Myopia is considered a major public health problem worldwide (1). By 2050, the number of individuals with myopia and high myopia is expected to reach 4.758 billion (prevalence, 49.8%) and 938 million (prevalence, 9.8%), respectively (2). Myopia and high myopia not only cause visual problems but also lead to serious complications, including macular degeneration and retinal detachment, which cause considerable social and economic burdens (3). Early onset myopia can result in severe myopia and subsequent complications (4). By 2050, the prevalence of myopia among children and adolescents in China will reach approximately 84% (5). Therefore, the early detection and prevention of myopia have become the focus of global research.

As children develop, their biometric parameters show varying degrees of change (6). Ocular biometric parameters are potential biomarkers for assessing myopia in children and adolescents (7). The axial length (AL) is a major determinant of myopia (8); the longer the AL, the more severe the myopia (9). Additionally, the AL to corneal radius of curvature (CR) ratio (AL/CR) may be used to evaluate myopia risk (10). The AL/CR ratio is significantly more effective for detecting myopia in children than either naked-eye vision or the AL and could be a useful marker for the onset and development of myopia (11).

Factors affecting myopia development are heritable and influenced by the environment (12, 13). As an endophenotype of myopia with a distinct genetic component (9), the AL/CR ratio is also associated with genetic factors (14). A survey of myopia-related factors in Japanese preschool children indicated that the AL and AL/CR ratio were associated with genetic and environmental factors (15).

Therefore, we aimed to elucidate which factors affect the ocular biological parameters of Chinese children. We conducted a survey to understand the effects of genetic, growth and development, and environmental factors on the ocular biometric parameters of preschool children in Beijing, China.

## 2 Methods

### 2.1 Participants

This cross-sectional study, involving a school-based cohort, began in November 2021 across nine kindergartens in the Tongzhou District, Beijing. The detailed methodology has been discussed previously (16). Overall, 1,917 children from two public and seven private kindergartens in Tongzhou District were selected using stratified random cluster sampling. The ocular biological parameters, height, and weight of each child were measured, and parents completed an online questionnaire on parental myopia and environmental influences. The AL/CR ratio were calculated. Children with obvious eye conditions, including glaucoma, cataracts, fundus disease, and strabismus, were excluded from this study. Participants who had difficulty following up or failed to cooperate with the questionnaire and examination were also excluded. Ultimately, 1,353 participants were enrolled in the study.

The study protocol conformed to the tenets of the Declaration of Helsinki, and the Ethics Committee of Beijing Tongren Hospital, affiliated with Capital Medical University, approved this study (TRECKY2020-152). Through online parent meetings, we described the purpose, methods, significance, and precautions of the project to the guardians of the participants, from whom informed consent was obtained.

### 2.2 Eye measurements

Ophthalmologists and nurses were involved in the study and received professional training before the examination. An ophthalmologist conducted the anterior segment and strabismus examinations. Ocular biometrics were measured in each eye using Lenstar 900 (Haag–Streit, Koeniz, Switzerland). The average of three measurements, automatically documented using the instrument, was recorded as the final result. If a significant deviation was observed in the results, the test was repeated. All participants were tested using the same machine.

The guardians of the participants completed an online questionnaire regarding genetic and environmental factors. Parental myopia was defined as biological parental myopia, and the exact degree was determined. Environmental factors included electronic product use, average daily outdoor time, and average daily continuous near-work time.

### 2.3 Definitions

Outdoor activities included exercise and rest. Near-work was defined as tasks performed within 33 cm of the eyes. Electronic products included mobile phones, computers, tablet computers, and game consoles. The average daily hours spent outdoors and performing continuous near-work were calculated using the following formula: [(hours spent on weekdays) × 5 + (hours spent on weekends) × 2]/7. Incomplete questionnaires were excluded from the analysis. The CR was determined as the average of the flattest and steepest radius. The AL/CR ratio was calculated as the AL divided by the CR. The correlation of the parameters of the two eyes was analyzed.

### 2.4 Statistical analysis

Data analysis was performed using R4.2.1 software. Ocular biological parameters are presented as the mean ± standard deviation. Due to the non-normal distribution and lack of variance homogeneity across different classification conditions of ocular biological parameters, the Mann–Whitney U test was used for binary variables. The Kruskal–Wallis test was used for variables with multiple classifications in the univariate analysis to explore the relationships between each risk factor and the AL, CR, and AL/CR ratio. We set the condition from univariate to multivariate. A value of 0.1 was also set to enter multivariate analysis. Subsequently, to understand the influence of parental myopia, environmental factors, sex adjustment, age, and other factors on the AL, CR, and AL/CR ratio, a multiple linear regression model was used. Statistical significance was set at *p* < 0.05.

## 3 Results

**Figure 1:**
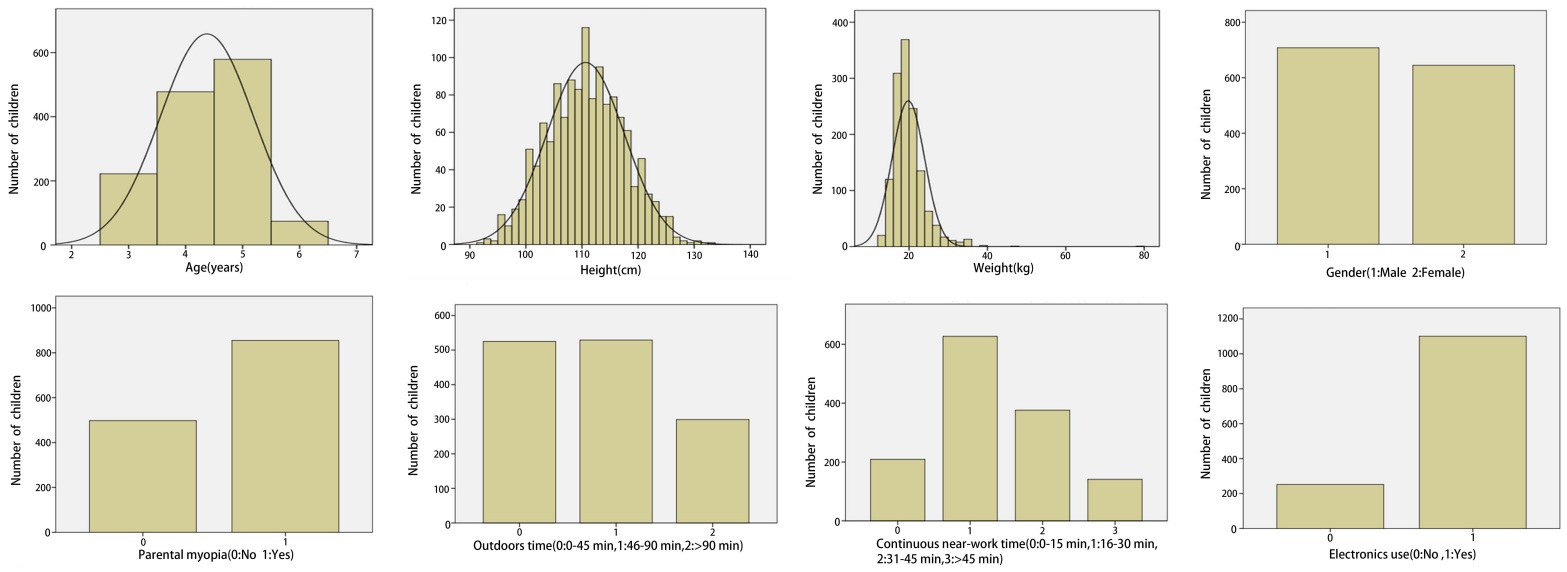
Data distribution density on demographics, parental myopia, growth and development, and environmental factors in preschool children.

Overall, 1,353 children participated in this study, including 708 (52.32%) boys and 645 (47.68%) girls. Their ages ranged from 3 to 6 years, with an average age of 4.37 ± 0.82 years. According to the questionnaire, 855 (63.19%) children had parents who had myopia, and 498 (36.81%) had parents who did not have myopia. Additionally, 1,101 (81.37%) children used electronic products. Regarding the daily time spent outdoors, 525 (38.80%), 529 (39.10%), and 299 (22.10%) children spent 0–45, 46–90, and >90 min outdoors, respectively. Concerning the daily time spent performing continuous near-work, 209 (15.45%), 627 (46.34%), 376 (27.79%), and 141 (10.42%) children spent 0–15, 16–30, 31–45, and >45 min, respectively. Furthermore, we found that the AL and CR of the left eyes were highly correlated with those of the right eyes (*r* = 0.953, *p* < 0.001; and *r* = 0.957, *p* < 0.001, respectively). We chose to analyze data from the right eyes. Data on the demographic characteristics of the participants, parental myopia prevalence, growth and development variables, and environmental factors are presented in Table 1. The distribution density of data is shown in Figure 1.

**Table 1:** Data on demographics, parental myopia, growth and development, and environmental factors in preschool children.

**Variable**	**Total (*n =* 1353)**
Age (years), mean ± SD	4.37 ± 0.82
Height (cm), mean ± SD	110.63 ± 6.93
Weight (kg), mean ± SD	19.81 ± 4.15
**Sex**, ***n*** **(%)**	
Male	708 (52.32)
Female	645 (47.68)
**Parental myopia**, ***n*** **(%)**	
No	498 (36.81)
Yes	855 (63.19)
**Electronics use**, ***n*** **(%)**	
No	252 (18.63)
Yes	1,101 (81.37)
**Outdoor time**, ***n*** **(%)**	
0–45 min	525 (38.80)
46–90 min	529 (39.10)
>90 min	299 (22.10)
**Continuous near-work time**, ***n*** **(%)**	
0–15 min	209 (15.45)
16–30 min	627 (46.34)
31–45 min	376 (27.79)
> 45 min	141 (10.42)

SD, standard deviation.

Univariate and multiple linear regression analyses of the impact of sex, age, parental myopia, and environmental factors on the AL are presented in Tables 2, 3. The univariate analysis revealed that longer ALs were significantly associated with older age (*p* < 0.001), male sex (*p* < 0.001), greater height (*p* < 0.001), higher weight (*p* < 0.001), and parental myopia (*p* = 0.006). The multiple linear regression model revealed that longer ALs were associated with male sex (*p* < 0.001), greater height (*p* < 0.001), and parental myopia (*p* = 0.002).

**Table 2:** Univariate analysis of AL-related factors in preschool children.

**Variables**	**β**	**S.E**	***t***	***P***	**β (95%CI)**
Age	0.21	0.02	9.12	<0.001	0.21 (0.16 −0.25)
**Gender**					
Male					0.00 (Reference)
Female	−0.50	0.04	−14.03	<0.001	−0.50 (−0.57 to −0.43)
**Parental myopia**					
No					0.00 (Reference)
Yes	0.11	0.04	2.77	0.006	0.11 (0.03–0.19)
**Outdoors time**					
0–45 min					0.00 (Reference)
46–90 min	0.07	0.04	1.55	0.121	0.07 (−0.02 to 0.15)
>90 min	0.07	0.05	1.31	0.191	0.07 (−0.03 to 0.17)
**Continuous near-work time**					
0–15 min					0.00 (Reference)
16–30 min	0.08	0.06	1.48	0.140	0.08 (−0.03 to 0.19)
31–45 min	0.09	0.06	1.47	0.143	0.09 (−0.03 to 0.21)
>45 min	0.09	0.08	1.19	0.232	0.09 (−0.06 to 0.24)
**Electronics use**					
No					0.00 (Reference)
Yes	0.04	0.05	0.81	0.419	0.04 (−0.06 to 0.14)
Height	0.04	0.00	14.54	<0.001	0.04 (0.03–0.04)
Weight	0.05	0.00	10.46	<0.001	0.05 (0.04–0.05)

CI, confidence interval; AL, axial length.

**Table 3:** Multivariate analysis of AL-related factors in preschool children.

**Variables**	**β**	**S.E**	***t***	***P***	**β (95%CI)**
Intercept	18.63	0.33	55.78	<0.001	18.63 (17.97–19.28)
Age	0.02	0.03	0.64	0.519	0.02 (−0.04–0.07)
**Gender**					
Male					0.00 (Reference)
Female	−0.46	0.03	−13.92	<0.001	−0.46 (−0.53 to −0.40)
**Parental myopia**					
No					0.00 (Reference)
Yes	0.11	0.03	3.11	0.002	0.11 (0.04–0.17)
Height	0.03	0.00	8.05	<0.001	0.03 (0.03–0.04)
Weight	−0.00	0.01	−0.01	0.989	−0.00 (−0.01 to 0.01)

CI, confidence interval.

Univariate and multiple linear regression analyses of the impact of sex, age, parental myopia, and environmental factors on the CR are presented in Tables 4, 5. The univariate analysis revealed that larger CRs were associated with male sex (*p* < 0.001), greater height (*p* < 0.001), higher weight (*p* < 0.001). The CR was significantly larger when the time spent outdoors exceeded 45 min (*p* = 0.023) and even more pronounced when it exceeded 90 min (*p* = 0.006). The multiple linear regression model revealed that larger CRs were associated with male sex (*p* < 0.001), greater height (*p* < 0.001). The CR was significantly larger when the time spent outdoors exceeded 90 min (*p* = 0.004).

**Table 4:** Univariate analysis of CR-related factors in preschool children.

**Variables**	**β**	**S.E**	***t***	***P***	**β (95%CI)**
Age	0.00	0.01	0.14	0.885	0.00 (−0.02 to 0.02)
**Gender**					
Male					0.00 (Reference)
Female	−0.13	0.01	−9.34	<0.001	−0.13 (−0.15 to −0.10)
**Parental myopia**					
No					0.00 (Reference)
Yes	0.01	0.01	0.37	0.713	0.01 (−0.02 to 0.03)
**Outdoors time**					
0–45 min					0.00 (Reference)
46–90 min	0.04	0.02	2.27	0.023	0.04 (0.01–0.07)
>90 min	0.05	0.02	2.77	0.006	0.05 (0.02–0.09)
**Continuous near-work time**					
0–15 min					0.00 (Reference)
16–30 min	0.02	0.02	1.07	0.283	0.02 (−0.02 to 0.06)
31–45 min	0.03	0.02	1.19	0.236	0.03 (−0.02 to 0.07)
>45 min	0.05	0.03	1.72	0.085	0.05 (−0.01 to 0.10)
**Electronics use**					
No					0.00 (Reference)
Yes	0.01	0.02	0.64	0.524	0.01 (−0.02 to 0.05)
Height	0.01	0.00	6.12	<0.001	0.01 (0.01–0.01)
Weight	0.01	0.00	4.56	<0.001	0.01 (0.01–0.01)

CI, confidence interval; CR, corneal radius of curvature.

**Table 5:** Multivariate analysis of CR-related factors in preschool children.

**Variables**	**β**	**S.E**	***t***	***P***	**β (95%CI)**
Intercept	7.16	0.12	57.71	<0.001	7.16 (6.92–7.40)
**Gender**					
Male					0.00 (Reference)
Female	−0.12	0.01	−9.00	<0.001	−0.12 (−0.15 to −0.10)
**Outdoors time**					
0–45 min					0.00 (Reference)
46–90 min	0.03	0.02	1.94	0.053	0.03 (−0.00 to 0.06)
>90 min	0.05	0.02	2.86	0.004	0.05 (0.02–0.09)
**Continuous near-work time**					
0–15 min					0.00 (Reference)
16–30 min	0.01	0.02	0.27	0.788	0.01 (−0.03 to 0.04)
31–45 min	0.01	0.02	0.35	0.724	0.01 (−0.03 to 0.05)
>45 min	0.03	0.03	1.28	0.201	0.03 (−0.02 to 0.09)
Height	0.01	0.00	4.40	<0.001	0.01 (0.01–0.01)
Weight	−0.00	0.00	−0.45	0.650	−0.00 (−0.01 to 0.00)

CI, confidence interval.

Univariate and multiple linear regression analyses of the impact of sex, age, parental myopia, and environmental factors on the AL/CR ratio are presented in Tables 6, 7. The univariate analysis revealed that higher AL/CR ratios were associated with older age (*p* < 0.001), male sex (*p* < 0.001), greater height (*p* < 0.001), higher weight (*p* < 0.001), and parental myopia (*p* = 0.002). When the time spent outdoors exceeded 90 min, the AL/CR ratio decreased (*p* = 0.035). The multiple linear regression model revealed that greater AL/CR ratios were significantly associated with older age, male sex, and parental myopia (all *p* < 0.001).

**Table 6:** Univariate analysis of AL/CR ratio-related factors in preschool children.

**Variables**	**β**	**S.E**	***t***	***P***	**β (95%CI)**
Age	0.03	0.00	11.90	<0.001	0.03 (0.02–0.03)
**Gender**					
Male					0.00 (Reference)
Female	−0.02	0.00	−4.62	<0.001	−0.02 (−0.02 to −0.01)
**Parental myopia**					
No					0.00 (Reference)
Yes	0.01	0.00	3.11	0.002	0.01 (0.01–0.02)
**Outdoors time**					
0–45 min					0.00 (Reference)
46–90 min	−0.00	0.00	−1.13	0.257	−0.00 (−0.01 to 0.00)
>90 min	−0.01	0.01	−2.12	0.035	−0.01 (−0.02 to −0.01)
**Continuous near-work time**					
0–15 min					0.00 (Reference)
16–30 min	0.00	0.01	0.47	0.639	0.00 (−0.01 to 0.01)
31–45 min	0.00	0.01	0.28	0.783	0.00 (−0.01 to 0.01)
>45 min	−0.01	0.01	−0.79	0.431	−0.01 (−0.02 to 0.01)
**Electronics use**					
No					0.00 (Reference)
Yes	0.00	0.00	0.17	0.869	0.00 (−0.01 to 0.01)
Height	0.01	0.00	9.57	<0.001	0.01 (0.01–0.01)
Weight	0.01	0.00	6.94	<0.001	0.01 (0.01–0.01)

CI, confidence interval; AL/CR ratio, axial length to corneal radius of curvature ratio.

**Table 7:** Multivariate analysis of AL/CR ratio-related factors in preschool children.

**Variables**	**β**	**S.E**	***t***	***P***	**β (95%CI)**
Intercept	2.72	0.04	75.96	<0.001	2.72 (2.65–2.79)
Age	0.02	0.00	7.97	<0.001	0.02 (0.02–0.03)
**Gender**					
Male					0.00 (Reference)
Female	−0.02	0.00	−4.71	<0.001	−0.02 (−0.02 to −0.01)
**Parental myopia**					
No					0.00 (Reference)
Yes	0.02	0.00	4.09	<0.001	0.02 (0.01–0.02)
**Outdoors time**					
0–45 min					0.00 (Reference)
46–90 min	−0.01	0.00	−1.61	0.109	−0.01 (−0.01 to 0.00)
>90 min	−0.01	0.00	−1.79	0.073	−0.01 (−0.02 to 0.00)
Height	0.00	0.00	0.46	0.642	0.00 (−0.00 to 0.00)
Weight	0.00	0.00	1.05	0.296	0.00 (−0.00 to 0.00)

CI, confidence interval.

## 4 Discussion

This study of kindergarten children demonstrated that ocular biological parameters are influenced by genetic, growth and development, and environmental factors. Multivariate analysis revealed that the AL, CR, and AL/CR ratio were greater in boys than in girls. Furthermore, the AL and CR were positively associated with height, while the AL/CR ratio increased with age. Parental myopia was associated with a significantly increased AL and AL/CR ratio, and the CR significantly increased when the daily time spent outdoors exceeded 90 min.

In this study, male sex and greater height were both associated with longer ALs and larger CRs in preschool children, consistent with the findings of previous studies (17–20). These results may be due to the coordinated growth between the eyes and body (21, 22). Zhang et al. (23) reported a significant association between the AL and height. In a 5-year cohort study, the AL was positively correlated with height; therefore, during periods of rapid height growth, more attention should be paid to the AL (21). Previous studies also showed that deeper vitreous cavities, longer ALs, and flatter corneas were observed in taller children (24–26). The AL and height elongation may be mediated partially by common genes (23). Hormone levels also affect the AL, and hormones associated with increased height can accelerate AL growth (27, 28). Therefore, the AL increases concurrently with height. Because boys were taller than girls between the ages of 3 and 6 years (29), they had longer ALs and greater CRs.

Additionally, we found that the AL/CR ratio was higher in males than in females, consistent with previous findings (17). Since most cases of juvenile myopia include axial myopia, AL elongation is considered the most crucial determinant of myopia incidence (30). When the AL is elongated to a certain extent, the CR is compensated, and the lens becomes thinner, focusing parallel light in front of the retina and preventing myopia (31). However, corneal flattening and lens thinning are limited (32–35). As boys develop faster than girls at this age, we speculate that the AL increases more rapidly than the CR; consequently, the AL/CR ratio will also be larger.

In this study, older children had higher AL/CR ratios; however, no significant correlation was found between the CR and age, although the CR increased slightly with age. The Anyang Childhood Eye Study (36) similarly found that 7-year-old children had CRs comparable to those of 14-year-old children. This may be because the corneal power has been shown to remain stable during the preschool stage (37). However, AL increased with age (38). Therefore, these studies collectively demonstrate that the AL/CR ratio increases with age. Our univariate analysis revealed a positive correlation between the AL and age, whereas the multiple linear regression model did not reveal this relationship. Moreover, previous study had demonstrated that older children have shorter CRs (39). Thus, more longitudinal studies are required to further investigate changes in the AL and CR with age during the preschool and school stages.

In our study, the AL and AL/CR ratio were strongly associated with parental myopia. Preschool children with parental myopia had longer ALs and higher AL/CR ratios (15). Klein et al. (40) reported that the refractive index and AL were highly heritable, and another study (9) showed that genes mainly determined the AL. Demir et al. (41) reported that Swedish children with two parents who had myopia had greater myopia and ALs than those with one or no parents with myopia. Edwards et al. (42) proposed that children with parents without myopia had smaller ALs than those with parents with myopia. A 2.5-year longitudinal cohort study (43) showed that children with parents with myopia had a faster rate of AL elongation, particularly those with two parents with myopia. A cohort study of cycloplegic refraction data from 9,793 children aged 6–72 months demonstrated that parental myopia, particularly in both parents, was associated with a greater risk of myopia, less hyperopic refraction, and a higher AL/CR ratio before school age (44). A study reported that when the AL/CR ratio was >2.99, children were more likely to have myopia (45). Therefore, eye development in children with parental myopia should be monitored as early as possible to prevent myopia.

In a 4-year follow-up study of Chinese primary school children in Beijing, greater axial elongation and larger increases in the AL/CR ratio were associated with less time spent outdoors (46). Comparably, a school-based cluster randomized trial showed that more time spent outdoors corresponded to less myopic shift and axial elongation (47). A meta-analysis of clinical studies reported that more hours of outdoor activity was associated with a slower increase in the AL (48). Our study found no significant relationship between the AL, AL/CR ratio, and time spent outdoors. However, this was a cross-sectional study; longitudinal follow-up studies should be conducted to observe the rate of AL, AL/CR ratio changes and determine the relationship between the time spent outdoors and AL, AL/CR ratio growth in preschool children in Beijing. In contrast, we found that the CR increased as the amount of time spent outdoors increased. While a previous study showed that corneal curvature was not associated with time spent outdoors (18). Longitudinally, observations of how the CR of Chinese preschool children changes with an increase in outdoor activity are necessary.

Using multiple linear regression models, we found no significant effects of body weight, continuous close-work time, and electronic use on the biological parameters of the eye. Wong et al. (49) reported that obesity does not affect eye growth. The link between screen time and myopia remains controversial. In one study, increased screen time (screen time >2 h per day) was associated with more myopic SER and a longer AL, shorter CR, and higher AL/CR ratio, while no association was found between ocular biometric parameters and reading time (50). Matsumura et al. (15) reported that a longer AL was significantly associated with screen time (>1 h per day on smartphones, tablets, and computers), while no significant correlation was found between the AL/CR ratio and screen time.

This study had some limitations. First, data on environmental and genetic factors were provided by parents and collected using a self-administered questionnaire, which may introduce recall bias. Second, this was a cross-sectional study, and we could not longitudinally assess how genetic, growth and development, and environmental factors contribute to alterations in ocular biological parameters. Third, we did not compare the effect of myopia severity in parents and the number of parents with myopia on the ocular biological parameters of children. Fourth, the outdoor light intensity was not quantified in this study. Fifth, although we found that genetics, outdoor time, height and other factors have a certain impact on children's eye biological parameters, but the child's other lifestyle, including diet, sleep, screen time, and other factors may also have different degrees of impact on their eye parameters. Therefore, we should have a more comprehensive understanding of children's life and eye habits. In addition, the study was based on data from Beijing, so its generalizability to other regions or countries may be limited. In the future, we will conduct longitudinal, in-depth, comprehensive research on the risk factors for ocular biological parameters.

## 5 Conclusions

Ocular biological parameters are influenced by genetic, growth and development, and environmental factors. Among children aged 3–6 years in Beijing, monitoring growth and development, investigating parental myopia, and intervening in eye habits have certain guiding significance for delaying the growth of the AL and AL/CR ratio. Our findings are expected to provide new ideas and new methods for the development of healthy eye habits, so that school, family and society can better supervise and develop habits for preschool children.

## Data Availability

The data used and/or analyzed during the current study are available from the corresponding author upon reasonable request. Requests to access the datasets should be directed to Bidan Zhu, zbd2017@qq.com.
